# Polymorphism in the Retinoic Acid Metabolizing Enzyme CYP26B1 and the Development of Crohn’s Disease

**DOI:** 10.1371/journal.pone.0072739

**Published:** 2013-08-19

**Authors:** Karin Fransén, Petra Franzén, Anders Magnuson, Ali Ateia Elmabsout, Nils Nyhlin, Anna Wickbom, Bengt Curman, Leif Törkvist, Mauro D’Amato, Johan Bohr, Curt Tysk, Allan Sirsjö, Jonas Halfvarson

**Affiliations:** 1 Department of Clinical Medicine, School of Health and Medical Sciences, Örebro University, Örebro, Sweden; 2 Clinical Epidemiology and Biostatistics Unit, Örebro University Hospital, Örebro, Sweden; Department of Internal Medicine, Division of Gastroenterology, Örebro University Hospital, Örebro, Sweden; 4 IBD Clinical research group, Karolinska University Hospital, Stockholm, Sweden; 5 Department for Clinical Science, Intervention and Technology, Karolinska Institutet, Stockholm Sweden; 6 Department of Biosciences and Nutrition, Karolinska Institutet, Stockholm, Sweden

## Abstract

Several studies suggest that Vitamin A may be involved in the pathogenesis of inflammatory bowel disease (IBD), but the mechanism is still unknown. Cytochrome P450 26 B1 (CYP26B1) is involved in the degradation of retinoic acid and the polymorphism rs2241057 has an elevated catabolic function of retinoic acid, why we hypothesized that the rs2241057 polymorphism may affect the risk of Crohn’s disease (CD) and Ulcerative Colitis (UC). DNA from 1378 IBD patients, divided into 871 patients with CD and 507 with UC, and 1205 healthy controls collected at Örebro University Hospital and Karolinska University Hospital were analyzed for the *CYP26B1* rs2241057 polymorphism with TaqMan® SNP Genotyping Assay followed by allelic discrimination analysis. A higher frequency of patients homozygous for the major (T) allele was associated with CD but not UC compared to the frequency found in healthy controls. A significant association between the major allele and non-stricturing, non-penetrating phenotype was evident for CD. However, the observed associations reached borderline significance only, after correcting for multiple testing. We suggest that homozygous carriers of the major (T) allele, relative to homozygous carriers of the minor (C) allele, of the *CYP26B1* polymorphism rs2241057 may have an increased risk for the development of CD, which possibly may be due to elevated levels of retinoic acid. Our data may support the role of Vitamin A in the pathophysiology of CD, but the exact mechanisms remain to be elucidated.

## Introduction

Inflammatory bowel disease (IBD) is the collective name of several chronic relapsing inflammatory disorders of the gastrointestinal tract, dominated by Crohn’s disease (CD) and ulcerative colitis (UC). These diseases are characterized by altered intestinal immune response against gut microbiota in genetically susceptible individuals. The precise pathogenesis is, however, still unclear. Previous studies have indicated on a relationship between the vitamin A analogue isotretinoin and the development of IBD [1], but the role of vitamin A in the development of IBD remains unclear. Vitamin A/retinol is metabolized in a two-step reaction into all-*trans*-Retinoic Acid (atRA), which is the most important bioactive metabolite. In the first step, vitamin A is reversibly oxidized to retinal by retinol dehydrogenases (RDHs), while step two irreversibly transforms retinal into atRA via retinal dehydrogenases (RALDHs) [2], [3]. The active metabolite, atRA, is an important molecule in many biological processes including maintenance of epithelial cells, regulation of apoptosis, embryogenesis and immune system surveillance [4]. AtRA is a ligand for nuclear retinoic acid receptors (RARα, RARβ or RARγ) and bind specifically to certain DNA elements (RAREs) throughout dimerization with retinoic X receptors (RXRα, RXRβ or RXRγ) [5], [6]. Generally, the RAREs are located in the promoter regions of downstream target genes for atRA [3], [7], [8]. One family of these target genes is the cytochrome P450 (*CYP*) genes, which is important for the elimination of atRA and thereby crucial for the control of the atRA homeostasis in the cell, both in the embryonic life as well as in the adult life [4]. Much of the atRA is likely degraded by CYP26A1, CYP26B1 and CYP26C1, where each CYP26 gene has specific expression patterns in the developing organism [3], [9]–[11]. Interestingly, CYP26B1 expression was previously found to be induced by atRA in T-cells of the mesenteric lymph nodes (MLN) and Peyer’s patches (PP) and changed expression of CYP26B1 may alter T cell trafficking and differentiation in the gut [12]. Kang and co-workers showed that both high and low vitamin A levels resulted in ameliorated intestinal inflammation and differentially induced subsets of FoxP3^+^ cells in SAMP1/YP mice [13].

Previously, a study from our group showed that the minor allele (C) of the rs2241057 polymorphism encoding a Leu to Ser amino acid shift in position 264 in the *CYP26B1* gene possessed enhanced retinoid catabolizing capacity [14]. Therefore, we hypothesized that the rs2241057 polymorphism in the *CYP26B1* gene may be involved in the pathogenesis of IBD. In the present study we aimed to investigate this polymorphism as a risk factor for the development of CD and UC, respectively, and to assess possible genotype-phenotype associations in a case-control study.

## Materials and Methods

### Ethics statement

Ethical approval was obtained from the regional ethical review boards of Karolinska Institutet, Stockholm, Sweden and Uppsala University, Uppsala Sweden. Written informed consent was obtained from IBD patients and verbal informed consent from healthy blood donors recruited at the Blood Bank of the Örebro University Hospital in accordance with the ethical approval. At arrival at the Blood Centre, blood donors were provided with written information, verbally informed about the study, and given the possibility to ask questions about the study. Exclusion criteria were history of any chronic gastrointestinal disease, including IBD. If a blood donor verbally approved to participate in the study, a blood sample was collected for the genetic analyses. This procedure was approved by the regional ethical board, only age and sex of the blood donors were recorded and there is no way to trace back identities of the included individuals. Furthermore, the ethical committee approved that the consent was not recorded in the medical notes, since the enrolment was anonymous. The ethical considerations for the study followed the principles of the Declaration of Helsinki.

### Study subjects

In total, 1378 Swedish patients with IBD, 871 with CD and 507 with UC, were recruited at Örebro University Hospital, Örebro and the Karolinska University Hospital, Stockholm. Diagnosis was based on standard clinical, endoscopic, radiologic and histologic criteria [15]. Medical notes were scrutinized to classify disease characteristics according the Montreal classification [16]. Data on clinical characteristics are presented in Table 1. Healthy blood donors without any history of gastrointestinal disease (n = 1205) were recruited from Örebro University Hospital as controls. The mean age of the controls was 43 years and the percentages of females and males were 43% and 57%, respectively.

**Table 1:** Clinical characteristics of patients with inflammatory bowel disease.

		UC (%)	CD (%)
**Number of subjects**		507	871
	Female	232 (46)	431 (49)
	Male	275 (54)	440 (51)
**Age at diagnosis** (mean ± SD)		34.5 ± 15.0	30.4 ± 14.8
**First-degree relative with IBD**		60 (14)	82 (14)
**Smoking habits**	Smoker	63 (13)	178 (27)
	Former smoker	175 (35)	163 (24)
	Never smoked	257 (52)	322 (49)
**Location at diagnosis**	L1 Ileal/+ Upper GI		140 (20)/+6 (1)
	L2 Colonic/+ Upper GI		333 (47)/+3 (0)
	L3 Ileocolonic/+ Upper GI		214 (30)/+7 (1)
	L4 Upper GI		4 (1)
**Behavior at diagnosis**	B1 Non-stricturing, non-penetrating		484 (69)
	B2 Stricturing		172 (24)
	B3 Penetrating		49 (7)
**Extent at diagnosis**	E1 Proctitis	133 (27)	
	E2 Left Sided colitis	159 (32)	
	E3 Extensive colitis	204 (41)	

CD =  Crohn’s disease, UC =  Ulcerative colitis.

### Genotyping of *CYP26B1* rs2241057 polymorphism

DNA was extracted from whole blood using QIAamp DNA Blood Maxi Kit (Qiagen, Hilden, Germany) according to the manufacturer’s instructions. For the genotyping of the SNP rs2241057 polymorphism in the *CYP26B1* gene, the TaqMan® SNP Genotyping Assay C_ _15872328_10 (Applied Biosystems, Foster City, CA) was used. The amplification mixtures contained 40 ng DNA, 1xTaqMan® Genotype PCR Master Mix (Applied Biosystems), 1xTaqMan® SNP Genotyping Assay (Applied Biosystems) including allele specific probe and primers labeled with the two allele specific fluorescent reporter dyes VIC (C allele) and FAM (T allele), and Milli-Q water in a 10μl reaction. The PCR reactions were performed on 7900HT Fast Real-Time PCR System (Applied Biosystems) using 96 well plates. The PCR profile was as follows: 10 min at 95°C and 40 cycles of 15 s at 95°C followed by 60 s at 60°C. Six negative controls were included on each plate. The amplification was followed by post-read allelic discrimination analysis.

### Statistics

The rs2241057 polymorphism was tested for Hardy-Weinberg equilibrium. Based on the assumptions of a CC genotype prevalence of 3.5% in the background population (http://www.ncbi.nlm.nih.gov/projects/SNP/snp_ref.cgi?rs=2241057), and an odds ratio of three in cases *vs*. controls, 656 patients with CD and UC, respectively, as well as 656 controls would be required to obtain a statistical power of 80% and a statistical significance level of 5%.

Statistical analysis was performed with the SPSS (SPSS Inc. Chicago, IL), Epi Info (CDC, Atlanta, GA) and STATA release 11 (Stata Corp LP, College Station, TX, USA) packages. Associations between categorical variable were assessed with Chi-square test or the Fisher’s exact test (two tailed) when appropriate. The data were presented as uncorrected *P-*values, odds ratio (OR) and 95% confidence interval (CI) as well as false discovery rate (FDR) corrected *P-*values, due to analyses of three groups, i.e. CD, UC and IBD. A *P*-value ≤ 0.05 was regarded as statistically significant.

## Results

### *CYP26B1* rs2241057 polymorphism in patients with Crohn’s disease and patients with ulcerative colitis

The genotypes for the patient and control populations were found in Hardy-Weinberg equilibrium and in similar genotype and allele frequencies as described in the NCBI SNP database for European population. A higher frequency of patients homozygous for the major (T) allele compared to the minor (C) allele in the *CYP26B1* rs2241057 polymorphism was observed in patients with CD compared with healthy controls for the *CYP26B1* rs2241057 polymorphism (OR =  2.2; CI 1.0–4.7; Table 2). This was also evident when comparing the frequency of heterozygotes (CT) with the frequency of CC individuals (OR =  2.1; CI 1.0–4.7; Table 2). However, when correcting for analyses of three different groups, i.e. CD, UC and the entire IBD population, statistical significance was not reached (Table 2). In contrast, significant associations were neither seen in patients with UC (OR =  1.0; CI 0.5–2.1; Table 2), nor in the total IBD population (OR =  1.5; CI 0.9–2.8; Table 2). The observed genotype associations could not be confirmed when comparing allele frequencies (Table 3).

**Table 2:** Genotype frequencies of the polymorphism rs2241057 in the *CYP26B1* gene in patients with inflammatory bowel disease *vs*. healthy controls.

	Genotype	Patients (%)	Controls (%)	OR	CI	*P* ^*^
**CD**	CC	11 (1)	28 (3)	1		
	CT	204 (25)	243 (25)	2.1	1.0–4.7	0.04/0.12
	TT	598 (74)	704 (72)	2.2	1.0–4.7	0.03/0.09
**UC**	CC	12 (3)	28 (3)	1		
	CT	110 (26)	243 (25)	1.1	0.5–2.3	0.88/0.88
	TT	295 (71)	704 (72)	1.0	0.5–2.1	0.91/0.91
**IBD**	CC	23 (2)	28 (3)	1		
	CT	314 (25)	243 (25)	1.6	0.9–2.9	0.12/0.18
	TT	893 (73)	704 (72)	1.5	0.9–2.8	0.13/0.20

Chi-square test used for *P*-values. Odds ratio and confidence interval estimated using 2×2 contingency tables. CD =  Crohn’s disease, UC =  Ulcerative colitis, C =  minor allele, T =  major allele. OR =  odds ratio, CI =  95% confidence interval. ^*^ Uncorrected P-value/FDR corrected P-value.

**Table 3:** Allele frequencies of the polymorphism rs2241057 in the *CYP26B1* gene in patients with inflammatory bowel disease *vs*. healthy controls.

	Allele	Patients (%)	Controls (%)	OR	CI	*P* ^*^
**CD**	C	226 (14)	299 (15)	1		
	T	1400 (86)	1651 (85)	1.1	0.9–1.4	0.23/0.34
**UC**	C	134 (16)	299 (15)	1		
	T	700 (84)	1651 (85)	0.95	0.8–1.2	0.62/0.62
**IBD**	C	360 (15)	299 (15)	1		
	T	2100 (85)	1651 (85)	1.1	0.9–1.3	0.52/0.78

Chi-square test used for *P*-values. Odds ratio and confidence interval estimated using 2×2 contingency tables. CD =  Crohn’s disease, UC =  Ulcerative colitis, C =  minor allele, T =  major allele. OR =  odds ratio, CI =  95% confidence interval.^ *^ Uncorrected P-value/FDR corrected P-value.

### Genotype-phenotype associations

The observed association between the TT genotype and CD remained when analyzing patients with non-stricturing, non-penetrating Crohn’s disease behavior only. A significant higher frequency of CD patients with non-stricturing, non-penetrating behavior were homozygous for the major allele (T), compared to homozygous carriers of the minor (C) allele (OR =  2.7; CI 1.0–8.0; Table 4), although the results could not be confirmed at allele frequency level (Table 5). Furthermore, an association between the T allele and young age at diagnosis of CD (A1) was revealed when comparing allele frequencies (OR = 1.8; CI 1.2–3.1; Table 5). Similarly, a trend towards higher frequency of the TT genotype *vs.* the CC genotype was observed in this subgroup of patients (*P* = 0.06; Table 4). Beyond the associations with non-stricturing, non-penetrating CD and young age at diagnosis, no genotype-phenotype associations were observed in any of the subgroups (Table 4, 5, S1 and S2).

**Table 4:** Genotype frequencies of the polymorphism *rs2241057* in the *CYP26B1* gene for patients with Crohn’s disease and healthy controls, displayed for sub phenotypes and clinical features.

		Genotype (%)			CT *vs*. CC			TT *vs*. CC		
		CC	CT	TT	OR	CI	*P*	OR	CI	*P*
**Male**	Controls	20 (3)	155 (27)	407 (70)	1			1		
	Patients	7 (2)	118 (28)	291 (70)	2.2	0.8–5.8	0.09	2.0	0.8–5.4	0.10
**Female**	Controls	8 (2)	88 (22)	297 (76)	1			1		
	Patients	4 (1)	86 (22)	307 (77)	2.0	0.5–8.1	0.28	2.1	0.6–8.2	0.23
**Controls Total**		28 (3)	243 (25)	704 (72)	1			1		
**Age at diagnosis**	A1 Age≤ 16 years	0 (0)	19 (17)	90 (83)	–	–	0.23^†^	–	–	0.06^†^
	A2 Age 17–40 years	7 (2)	106 (27)	272 (71)	1.7	0.7–4.1	0.20	1.5	0.7–3.6	0.31
	A3 Age> 40 years	1 (1)	36 (22)	125 (77)	4.1	0.5–31.4	0.23^†^	5.0	0.7–36.9	0.10^†^
Location at diagnosis^*^	L1 Ileal	1 (1)	30 (24)	94 (75)	3.5	0.5–70.7	0.33^†^	3.7	0.5–74.7	0.24^†^
	L2 Colonic	5 (2)	69 (22)	233 (76)	1.6	0.6–4.9	0.35	1.9	0.7–5.5	0.20
	L3 Ileocolonic	2 (1)	53 (27)	143 (72)	3.1	0.7–19.1	0.12	2.8	0.7–17.5	0.21^†^
	L4 Upper GI	0 (0)	0 (0)	4 (100)	–	–	–	–	–	1.00^†^
Behavior at diagnosis^*^	B1 Non-stricturing, non-penetrating	5 (1)	109 (24)	336 (75)	2.5	0.9–7.6	0.06	2.7	1.0–8.0	0.04
	B2 Stricturing	2 (1)	38 (24)	116 (75)	2.2	0.5–13.9	0.40^†^	2.3	0.5–14.2	0.42^†^
	B3 Penetrating	1 (2)	10 (24)	31 (74)	1.1	0.1–24.9	1.00^†^	1.2	0.2–25.1	1.00^†^

Chi-square test used for *P*-values unless otherwise stated. Odds ratio and confidence interval estimated using 2×2 contingency tables. C =  minor allele, T =  major allele. OR =  odds ratio, CI =  95% confidence interval. ^*^Patients with combination of two locations are excluded in this overview, ^†^ Fisher’s two tailed exact test used.

**Table 5:** Allele frequencies of the polymorphism *rs2241057* in the *CYP26B1* gene for patients with Crohn’s disease versus healthy controls, displayed for sub phenotypes and clinical features.

		Allele frequencies (%)				
		C	T	OR	CI	*P*
**Male**	Controls	195 (17)	969 (83)	1		
	Patients	132 (16)	700 (84)	1.1	0.8–1.4	0.60
**Female**	Controls	104 (13)	682 (87)	1		
	Patients	94 (12)	700 (88)	1.1	0.6–1.6	0.40
**Controls Total**		299 (15)	1651 (85)	1		
**Age at diagnosis**	A1 Age ≤16 years	19 (9)	199 (91)	1.8	1.2–3.1	<0.01
	A2 Age 17–40 years	120 (16)	650 (84)	1.0	0.8–1.2	0.87
	A3 Age >40 years	38 (12)	286 (88)	1.4	1.0–2.0	0.09
Location at diagnosis^*^	L1 Ileal	32 (13)	218 (87)	1.2	0.8–1.9	0.29
	L2 Colonic	79 (13)	535 (87)	1.2	0.9–1.6	0.13
	L3 Ileocolonic	57 (14)	339 (86)	1.1	0.8–1.5	0.63
	L4 Upper GI	0 (0)	8 (100)	–	–	0.61^†^
Behavior at diagnosis^*^	B1 Non-stricturing, non-penetrating	119 (13)	781 (87)	1.2	0.9–1.5	0.14
	B2 Stricturing	42 (13)	270 (87)	1.2	0.8–1.7	0.39
	B3 Penetrating	12 (14)	72 (86)	1.1	0.6–2.1	0.79

Chi-square test used for *P*-values unless otherwise stated. Odds ratio and confidence interval estimated using 2×2 contingency tables. C =  minor allele, T =  major allele. OR =  odds ratio, CI =  95% confidence interval. ^*^Patients with combination of two locations are excluded in this overview, ^†^ Fisher’s two tailed exact test used.

## Discussion

IBD, including CD and UC, is an idiopathic condition with chronic inflammation of the gut. Several pieces of evidence support the role of the Vitamin A in the pathophysiology of IBD. AtRA, the most bioactive metabolite of Vitamin A/retinol, is mainly regulated by CYP26 proteins. Our findings of a possible association between the *CYP26B1* rs2241057 polymorphism influencing retinoid catabolism, and CD may support the role of Vitamin A in IBD. Homozygous genotype of the major (T) allele seems to be a possible risk factor for the development of CD. We have previously shown that the minor (C) allele catabolizes retinoic acid more efficient than the major allele [14]. Thus, the TT genotype in *CYP26B1* rs2241057 is probably associated with higher levels of retinoic acid, due to the lower catabolic activity, which may result in an increased risk of CD. Our results are supported by the observed risk of IBD in patients treated with the vitamin A analogue isotretinoin (13-*cis*-retinoic acid) for severe acne [17], [18]. However, the association between the Vitamin A analogue isotretinoin and IBD has been questioned [19] and Crockett and coworkers observed an association between isotretinoin exposure, including a dose-response-relationship, and UC only [1].

Despite increasing data, the role of retinoids in IBD is still controversial. Contradictory to our findings, Reifen *et al*. showed that Vitamin A deficiency induces colonic inflammation [20]. Furthermore, it has been proposed that vitamin A down-regulates the intestinal inflammation in patients with ulcerative colitis as well as *in vitro* in murine colitis [21]. On the other hand, Kang *et al*. showed that both limited and excessive vitamin A conditions induced distinct FoxP3(+) T-cell subsets, and both ameliorated the intestinal inflammation in mice [13]. It has also been suggested that atRA induces chemokine receptor 9 (CCR9) in T-cells in a CYP26B1 dependent manner [12]. Based on data from mice model, CCR9 seems to induce migration of Tregs to the intestine and administration of antibodies against CCR9 in tumor necrosis factor Δadenosine uracil-rich element mice exacerbated ileitis [22]. Thus, one could speculate if homozygous carriage of the major (T) allele would be associated with diminished inflammation in the gut. On the other hand, recent trials examining the efficacy of CCX282-B, which targets the CCR9 receptor, have shown promising results both for induction and maintenance of remission in patients with CD [23].

CD has historically been associated with a T_h_1-type T-cell mediated inflammation, but in more recent years the role of IL-17 secreting T_h_17 cells have been discovered [24]. AtRA has been shown to be important both for induction and homing of Th17 cells [25] and mice fed with vitamin A-free diet had lower frequency of T_h_17 cells [26]. Interestingly, the observed associations with the *CYP26B1* rs2241057 polymorphism were significant for CD patients with inflammatory behavior or young age at diagnosis, and not for patients with the other sub-phenotypes of CD. It can be speculated if the observed association between the *CYP26B1* rs2241057 polymorphism and CD is mediated by changes in the atRA metabolism and thereby altered induction and homing of Th17 cells within the IL-23/Th17 pathway.

The fact that our findings could not be confirmed when comparing allele frequencies is a limitation of the study. However, based on the biologic driven hypothesis and previous clinical observations in vitamin A treated IBD patients, we aimed to detect differences in genotype. A study design powered to demonstrate differences in allele frequencies would require an enormous number of cases and controls. Our findings are also limited by the observation that the associations in CD were not significant, after correcting for analyses of three subgroups, i.e. CD, UC and the entire IBD cohort. However, the study was not powered to detect significant differences after correction for analyses of CD, UC and the total IBD population. It can not be excluded that absence of significant associations in UC is explained by the lower number of enrolled patients with UC, although some genetic variants seem to be disease specific and others are shared by both CD and UC [27]. Another possible limitation of the study is the lack of standardization in terms of ethnicity. Yet, patients and controls were recruited from a fairly homogenous Caucasian population within a defined geographic area, with a minor contribution of non-Caucasian patients and controls. Overall, these limitations underscore that our observations should be replicated in other Caucasian cohorts and in a second step also in non-Caucasian populations.

In conclusion, we observed a possible association between homozygous carriers of the major (T) allele of the *CYP26B1* polymorphism rs2241057 and CD. Since the *CYP26B1* gene is one of the major regulators of vitamin A our data supports the role of vitamin A in CD. Further replication efforts and mechanistic studies exploring the influence of vitamin A and *CYP26B1* in the pathogenesis of CD are warranted.

## Supporting Information

Table S1 **Genotype frequencies of the polymorphism rs2241057 in the** ***CYP26B1*** **gene for patients with Ulcerative colitis and healthy controls, displayed for sub phenotypes and clinical features.**
(DOCX)

Table S2 **Allele frequencies of the polymorphism rs2241057 in the** ***CYP26B1*** **gene for patients with Ulcerative colitis and healthy controls, displayed for sub phenotypes and clinical features.**
(DOCX)
