# Supplementary material for: Polymorphism in the Retinoic Acid Metabolizing Enzyme CYP26B1 and the Development of Crohn’s Disease
Source: PLoS One. 2013 Aug 19;8(8):e72739. doi: 10.1371/journal.pone.0072739 (PMC3747106; doi:10.1371/journal.pone.0072739)
Supplement: Table S1 — Genotype frequencies of the polymorphism rs2241057 in the CYP26B1 gene for patients with Ulcerative colitis and healthy controls, displayed for sub phenotypes and clinical features. (DOCX) [file pone.0072739.s001.docx]

| **Table S1.** Genotype frequencies of the polymorphism rs2241057 in the *CYP26B1* gene for patients with Ulcerative colitis and healthy controls, displayed for sub phenotypes and clinical features. C=minor allele, T=major allele. Chi-square test used for *P*-values unless otherwise stated. | | | | | | | | | | |
| --- | --- | --- | --- | --- | --- | --- | --- | --- | --- | --- |
|  | | **Genotype (%)** | | | **CT *vs.* CC** | | | **TT *vs.* CC** | | |
|  | | **CC** | **CT** | **TT** | **OR** | **CI** | ***P*** | **OR** | **CI** | ***P*** |
| **Male** | Controls | 20 (3) | 155 (27) | 407 (70) | 1 |  |  | 1 |  |  |
|  | Patients | 9 (4) | 64 (27) | 162 (69) | 0.9 | 0.4-2.3 | 0.84 | 0.9 | 0.4-2.2 | 0.77 |
| **Female** | Controls | 8 (2) | 88 (22) | 297 (76) | 1 |  |  | 1 |  |  |
|  | Patients | 3 (2) | 46 (25) | 133 (73) | 1.4 | 0.3-7.0 | 0.75^*^ | 1.2 | 0.3-5.8 | 1.00^*^ |
| **Controls Total** | | 28 (3) | 243(25) | 704 (72) | 1 |  |  | 1 |  |  |
| **Extent at diagnosis** | E1 Proctitis | 3 (3) | 29 (27) | 76 (70) | 1.1 | 0.3-4.9 | 1.00^*^ | 1.0 | 0.3-4.3 | 1.00^*^ |
|  | E2 Left sided colitis | 2 (1) | 30 (22) | 104 (77) | 1.7 | 0.4-11.1 | 0.75^*^ | 2.1 | 0.5-12.8 | 0.41^*^ |
|  | E3 Extensive colitis | 6 (4) | 48 (29) | 110 (67) | 0.9 | 0.3-2.6 | 0.86 | 0.7 | 0.3-2.0 | 0.45^*^ |

Chi-square test used for *P*-values unless otherwise stated. Odds ratio and confidence interval estimated using 2x2 contingency tables.
C= minor allele, T= major allele. OR= odds ratio, CI= 95% confidence interval. *Fisher’s two tailed exact test used.
