# Supplementary material for: Polymorphism in the Retinoic Acid Metabolizing Enzyme CYP26B1 and the Development of Crohn’s Disease
Source: PLoS One. 2013 Aug 19;8(8):e72739. doi: 10.1371/journal.pone.0072739 (PMC3747106; doi:10.1371/journal.pone.0072739)
Supplement: Table S2 — Allele frequencies of the polymorphism rs2241057 in the CYP26B1 gene for patients with Ulcerative colitis and healthy controls, displayed for sub phenotypes and clinical features. (DOCX) [file pone.0072739.s002.docx]

| **Table S2.** Allele frequencies of the polymorphism rs2241057 in the *CYP26B1* gene for patients with Ulcerative colitis and healthy controls, displayed for sub phenotypes and clinical features. | | | | | | |
| --- | --- | --- | --- | --- | --- | --- |
|  | | **Allele frequencies (%)** | |  |  |  |
|  | | **C** | **T** | **OR** | **CI** | ***P*** |
| **Male** | Controls | 195 (17) | 969 (83) | 1 |  |  |
|  | Patients | 82 (17) | 388 (83) | 0.9 | 0.7-1.3 | 0.73 |
| **Female** | Controls | 104 (13) | 682 (87) | 1 |  |  |
|  | Patients | 52 (14) | 312 (86) | 0.9 | 0.6-1.3 | 0.63 |
| **Controls Total** | | 299 (15) | 1651 (85) | 1 |  |  |
| **Extent at diagnosis** | E1 Proctitis | 35 (16) | 181 (84) | 0.9 | 0.6-1.4 | 0.74 |
|  | E2 Left sided colitis | 34 (12) | 238 (88) | 1.3 | 0.9-1.9 | 0.22 |
|  | E3 Extensive colitis | 60 (18) | 268 (82) | 0.8 | 0.6-1.1 | 0.17 |

Chi-square test used for *P*-values unless otherwise stated. Odds ratio and confidence interval estimated using 2x2 contingency tables.
C= minor allele, T= major allele. OR= odds ratio, CI= 95% confidence interval.
